# Distinguishing groups and exploring health differences among multiple job holders aged 45 years and older

**DOI:** 10.1007/s00420-018-1351-2

**Published:** 2018-09-08

**Authors:** Stef Bouwhuis, Trynke Hoekstra, Paulien M. Bongers, Cécile R. L. Boot, Goedele A. Geuskens, Allard J. van der Beek

**Affiliations:** 1 Department of Public and Occupational Health, Amsterdam UMC, Vrije Universiteit Amsterdam, Amsterdam Public Health research institute, Amsterdam, The Netherlands; 2 Netherlands Organisation for Applied Scientific Research TNO, Leiden, The Netherlands; 3 Body@Work, Research Center on Physical Activity, Work and Health, TNO-VU/VUmc, Amsterdam, The Netherlands; 4 Department of Health Sciences, Faculty of Science, Vrije Universiteit Amsterdam, Amsterdam, The Netherlands; 5 Department of Epidemiology and Biostatistics, Amsterdam UMC, Vrije Universiteit Amsterdam, Amsterdam Public Health research institute, Amsterdam, The Netherlands

**Keywords:** Moonlighting, Latent class analysis, Self-perceived health, Aging employee

## Abstract

**Purpose:**

To identify distinct groups of older multiple job holders and to explore health differences between these groups.

**Methods:**

We selected respondents from STREAM, a Dutch cohort study among persons aged 45 years and older, who reported having multiple jobs (*N* = 702). We applied latent class analysis to identify groups of multiple job holders. The association between these groups and health, measured with the SF-12, was studied cross-sectionally and longitudinally (1 year of follow-up), using linear regression analyses.

**Results:**

Four groups of older multiple job holders were identified: (1) a vulnerable group (*N* = 145), who preferred having one job, and had jobs with high demands and low resources; (2) an indifferent group (*N* = 134), who did not experience many benefits or disadvantages of multiple job holding (MJH); (3) a satisfied hybrid group, who were all self-employed in their second job (*N* = 310); and (4) a satisfied combination group, who all had a second job as an employee (*N* = 113). Both the satisfied hybrid and satisfied combination groups preferred MJH and experienced benefits of it. At baseline, the vulnerable group experienced significantly lower physical and mental health than the other groups. We found no significant differences regarding changes in health after 1 year.

**Conclusions:**

Four groups of older multiple job holders could be distinguished. The vulnerable group experienced lower physical and mental health at baseline than the other three groups. Policies and interventions supporting vulnerable multiple job holders may need to be developed. Future research is recommended to take heterogeneity among multiple job holders into account.

## Introduction

Over the last decades, the standard employment relation (SER), characterized by full-time stable employment as well as social rights and protection, has become less common in many countries (Van Aerden et al. 2016). A negative association between characteristics of non-SER employment relations, particularly job insecurity and temporary contracts, and health has been widely documented (De Witte et al. 2016; Van Aerden et al. 2016). Multiple job holding (MJH), meaning having multiple paid jobs, is an underresearched, non-standard working pattern (Panos et al. 2014). MJH is most prevalent in Northwest European countries. In Iceland, 12% of the working population had multiple jobs in 2017, and in Norway, Sweden, Denmark, and The Netherlands around 8% (Eurostat 2018). In the US, around 5% of the working population reported having multiple jobs (U.S. Bureau of Labor Statistics 2018).

Previous research has suggested that distinct groups of multiple job holders may exist. First, multiple job holders have been shown to be a heterogeneous group of workers, e.g., regarding demographic background, job characteristics, and reasons for having multiple jobs (Hipple 2010; Bamberry and Campbell 2012; Dorenbosch et al. 2015). Second, two studies have suggested that (adverse) characteristics of multiple job holders may cluster in groups. A recent qualitative study found three groups of multiple job holders, one of which mainly experienced advantages of MJH. Another group mainly experienced disadvantages and the third group did experienced advantages nor disadvantages (Bouwhuis et al. 2018). Another study has conceptualized, but not empirically studied, profiles of multiple job holders. This study proposed the bulimic profile (consisting of highly educated workers who voluntarily combine two full-time jobs), the cautious entrepreneur profile, the proletarian survivor profile, and the ideal-type futuristic profile (characterized by voluntary MJH with a favorable combination of jobs) (Rouault 2002). These two studies have suggested characteristics that may cluster in different groups of multiple job holders, i.e., reasons for MJH, experiences with MJH, job characteristics, and personal context. Having multiple jobs out of financial necessity seems to cluster with negative experiences with MJH, not being satisfied with the jobs (Bouwhuis et al. 2018), a lack of  personal resources to change their situation such as qualifications, self-efficacy, and finances (Rouault 2002), and a lack of a personal support system such as a partner (Bouwhuis et al. 2018).

Health differences between groups of multiple job holders may exist. Cross-sectional research has shown that those who have multiple jobs out of financial necessity experience lower mental health compared to workers who have multiple jobs for other reasons (Dorenbosch et al. 2015). The aspiration and deprivation hypotheses suggest that those employees who are more energetic and more ambitious, as well as those who are economically deprived and socially withdrawn are more likely to become multiple job holder, respectively (Jamal 1998). Health differences between groups of multiple job holders may thus already be apparent at the onset of MJH. In addition, different groups of multiple job holders may experience different health consequences, for instance, because differences in job characteristics between these groups. Conflicts between work schedules and job roles may result in stress, for some multiple job holders, for instance (Bamberry and Campbell 2012). Long working hours may result in less sleep and less leisure time, which may negatively affect health (Marucci-Wellman et al. 2014a, b, 2016). Therefore, health differences between groups may change over time.

Although the previous research has suggested that health consequences of MJH may differ among multiple job holders, no studies as of yet have empirically researched whether or not distinct groups of multiple job holders can be identified, and whether or not these groups differ regarding health. Insight in such groups and health differences between them is important, because it will provide knowledge regarding for which groups MJH has positive consequences regarding health and sustainable employability, and for which groups it has negative consequences. This may in turn contribute to the development of policies or interventions improving sustainable employability of multiple job holders. This is especially important to older multiple job holders, since the statutory retirement age is increasing in many countries, and because MJH could both improve or deteriorate sustainable employability. Therefore, the main research question of the present study is: which distinct groups can be identified among multiple job holders aged 45 years and older? In addition, we will explore whether distinct groups of multiple job holders differ regarding health.

## Methods

### Study population and design

The study population consisted of participants in STREAM, the Study on Transitions in Employment, Ability and Motivation (Ybema et al. 2014). STREAM is a longitudinal study that started in 2010. Persons who participated in an existing online panel of a Dutch market research company aged 45–64 years were invited to participate at baseline (*N* = 26,601). They were stratified by 5-year age groups and occupational status (i.e., employed, self-employed, and not employed). At baseline, 15,118 persons participated, among which 12,055 employees, 1029 self-employed persons, and 2034 not-working persons. They filled out online questionnaires annually (except 2014) on work and health, among other things. In 2015, a new cohort (*N* = 6738) was added to the existing 2010 cohort, to add persons aged 45–49 years again, and to compensate for loss to follow-up in the different age categories. In this study, we included participants of STREAM who reported having multiple jobs in the fifth measurement (2015). Some of these participants had been participating in STREAM since the baseline measurement (2010), while others were newly included when the new cohort was added in 2015. We chose to include participants from the fifth measurement, because in 2015, questions on reasons for and experiences with MJH were added to the STREAM questionnaire.

### Multiple job holding

MJH was measured with one question. Respondents indicated whether they: (1) had one job as an employee; (2) had multiple jobs as an employee; (3) were self-employed; (4) were unemployed; (5) were work disabled; (6) retired early; (7) retired; (8) were receiving education; or (9) were a home maker. In line with the previous research, we defined MJH as either having multiple jobs as an employee, or working as an employee and being self-employed (Bouwhuis et al. 2017a).

### Variables used to identify groups

The selection of variables used to identify groups of multiple job holders was based on the literature on MJH (Bamberry and Campbell 2012; Dorenbosch et al. 2015a, b; Hipple 2010; Wu et al. 2009; Bouwhuis et al. 2017a). We included variables in the following domains: reasons for MJH, satisfaction with work and MJH, work characteristics, ability to change life and work, social factors, and financial factors. In Table 1, an overview of the variables included is presented.

**Table 1:** Overview of the variables used to identify groups

Variable	Description	Measurement	Scale/categories
Reasons for MJH			
Reason for MJH	Most important reason why a respondent has multiple jobs	One question, based on the previous studies. Hipple (2010), Bamberry and Campbell (2012) and Koppes et al. (2011)	0. Impossible to work more hours in current job 1. Work more hours to make ends meet 2. To earn some extra money 3. To retain income security 4. To start a business 5. To get experience in another job 6. Because of the variation 7. Because I enjoy the combination of jobs
Pro-active development	Extent to which respondents actively pursue activities to keep their knowledge and skills up-to-date	Scale, consisting of four questions, based on Van Veldhoven et al. (2008)	Continuous (1–5)
Satisfaction with work and MJH			
Job satisfaction	Extent to which respondents are satisfied with their work, all things considered	One question, based on national working conditions survey. Koppes et al. (2011)	0. Unsatisfied 1. Unsatisfied nor satisfied 2. Satisfied
MJH history	Number of years a respondent has had multiple jobs	One question asking since which year respondents had multiple jobs	Continuous
Experience with MJH	Questions on experiences with MJH 1. Extent to which combining work schedules provides respondents with freedom 2. Extent to which combining work schedules provides respondents with stress 3. Extent to which demands of both employers are difficult to combine 4. Extent to which a respondent does tasks for one job while they are working in another job 5. Extent to which a respondent learns skills in one job that they can use in another job 6. Extent to which respondents perform tasks in one job better due to the other job 7. Extent to which respondents perform tasks in one job worse due to the other job	Seven questions, based on the previous research. Bamberry and Campbell (2012)	0. No 1. Yes For 2, 3, 4, 6, and 7: No: disagree Yes: do not disagree/do not agree; and agree For 1, 5: No: Disagree; and do not disagree/do not agree Yes: Agree Dichotomized based on the distribution of answers in the original variables
Rather a single job?	Would respondent prefer a single job to multiple jobs?	One question	0. No 1. Yes
Work characteristics			
Type of MJH	Multiple jobs as an employee (combination MJH) or jobs as an employee and self-employed (hybrid MJH)?	One question	0. Combination MJH 1. Hybrid MJH
Contract type	Does respondent have a permanent contract, or another contract type? Available for first and second job	Three questions on contract type, based on national working conditions survey. Koppes et al. (2011)	0. No permanent contract 1. Permanent contract in one job 2. Permanent contract in both jobs
Total working hours	Combination of total working hours (including overtime) in first and second job	Two questions, based on national working conditions survey. Koppes et al. (2011)	Continuous
Working in evenings/weekends	Combination of two questions, on evening/night work, and on weekend work	Two questions, based on national working conditions survey. Koppes et al. (2011)	0. No 1. Yes
Job demands and resources	1. Physical demands 2. Quantitative job demands 3. Autonomy All measured for first and second job	1. Quantitative job demands 2. Physical demands 3. Autonomy. Karasek (1985), Hildebrandt et al. (2010) and Kristensen et al. (2005)	For each demand/resource: 0. Low in both jobs 1. Low in one job; high in other 2. High in both jobs
Ability to change life and work			
Mastery	Extent to which respondents feel they are in control of life events	Pearlin Mastery scale. Pearlin et al. (1981)	Continuous (1–5)
Ability to find new employer	Extent to which respondents feel they are able to find a new employer within the next 12 months	One question	0. No 1. Maybe 2. Yes
Social factors			
Informal care	Whether or not a respondent provides informal care	One question, based on ‘OSA aanbodspanel study.’ OSA/SCP (2017)	0. No 1. Yes
Having a partner	Does respondent have a partner who lives with them?	One question	0. No 1. Yes
Work-home interference	Extent to which respondents’ work interferes with private life	One question based on Fox en Dwyer. Fox and Dwyer (1999)	0. No/seldom 1. Yes, often
Financial factors			
Financial situation household	Is the respondent’s household short of money, can it just come by or does it have money left?	One question, based on ‘OSA aanbodspanel study’. OSA/SCP (2017)	0. Short of money 1. Just enough 2. Money left
Breadwinner	Does the respondent provide the main household income?	One question, based on European Working Conditions survey. Parent-Thirion (2007)	0. No 1. Yes

### Outcome measure

To measure health, we used the 12-item short-form health survey (SF-12) (Ware et al. 1996). The SF-12 measures self-perceived health in the following eight domains: physical function, role limitations due to physical problems, bodily pain, general health, vitality, social function, role limitations due to emotional problems, and mental health. It includes questions, such as “In general, would you say your health is … [Excellent(1)-Poor(5)]”. We constructed separate variables for the physical functioning scores and the mental functioning scores. Both variables were standardized using the USA 1998 standards, resulting in a range from 0 to 100. A higher score reflected better physical or mental health.

### Analyses

To distinguish groups of multiple job holders, latent class analysis (LCA) was performed using MPlus 7.11. Our approach to LCA was based on the approach described by Jung and Wickrama (2008). To determine the optimal number of classes *k*, i.e., groups of multiple job holders, the first step was to specify a single-class model. Second, multi-class models were specified. In the third step, the best model was chosen using criteria relating to model fit, usefulness, and interpretability.

To determine model fit, several statistical parameters were used. First, we used the Bayesian information criterion (BIC), because previous modeling studies have suggested that the BIC outperforms other information criteria, e.g., the Akaike Information Criterion (AIC) (Nylund et al. 2007). The BIC takes into account the likelihood of the model and the number of parameters to determine model fit. A lower BIC indicates better model fit. Second, we used the Bootstrapped Likelihood Ratio Test (BLRT) to determine whether the *k* classes solution was a significant improvement over the *k*−1 classes solution. Previous research has shown that the BLRT outperforms other likelihood ratio tests (LRT), e.g., the Lo-Mendel-Rubin LRT (Nylund et al. 2007). Third, the average posterior probabilities in each of the subgroups were taken into account to determine how well respondents are classified in the classes (Hoekstra 2013). Although Yung and Wickrama did not specify a cut-off value, they stated the posterior probabilities should be high (close to 1.0) (Jung and Wickrama 2008). Other research has suggested to use a cut-off point of 0.8 (Clark and Muthén 2009), which we have followed.

It has been recommended to not only use statistical parameters to choose the best model (Jung and Wickrama 2008; Muthén 2003). Therefore, we also applied criteria related to usefulness and interpretability. Usefulness refers to the extent to which a *k* classes solution has enough participants in each class to be useful in further analysis. Usually, a solution in which each of the classes contains at least 1% of the study population is considered useful (Jung and Wickrama 2008). Because of the size of our study population, we have chosen to use 5% as a threshold for usefulness, which equals a minimum of *N* = 35 respondents in each class. Interpretability is related to the extent to which individual classes and the overall *k* classes solution are interpretable in light of earlier research or hypotheses on groups of multiple job holders (Bouwhuis et al. 2018; Rouault 2002).

Respondents were classified in the group which best suited them, based on the individual posterior probabilities. The groups were labeled based on variables that were most distinctive of that group. To gain more insight into the identified groups, we analyzed whether significant differences between these groups existed regarding gender, age, educational level, and economic sector using *χ*^2^-tests.

To explore whether groups of multiple job holders differ regarding health, we used linear regression analyses. We analyzed health differences at baseline (2015) as well as differences in changes in health after 1 year of follow-up (2016). Regarding differences in health at baseline, we performed crude analyses, in which the groups of MJH were the independent variable and physical and mental health were the outcome measures. For each of the outcome measures, a separate model was constructed. In addition, adjusted analyses were performed, in which age, gender, and educational level were included. To study differences in changes in health, identical models were used, with the exception that health at baseline was added as an independent variable to these models in the crude and adjusted analyses. Effect modification was tested by adding interaction terms for MJH group × gender and MJH group × age (45–54 years versus 55 years and older).

## Results

**Fig. 1:**
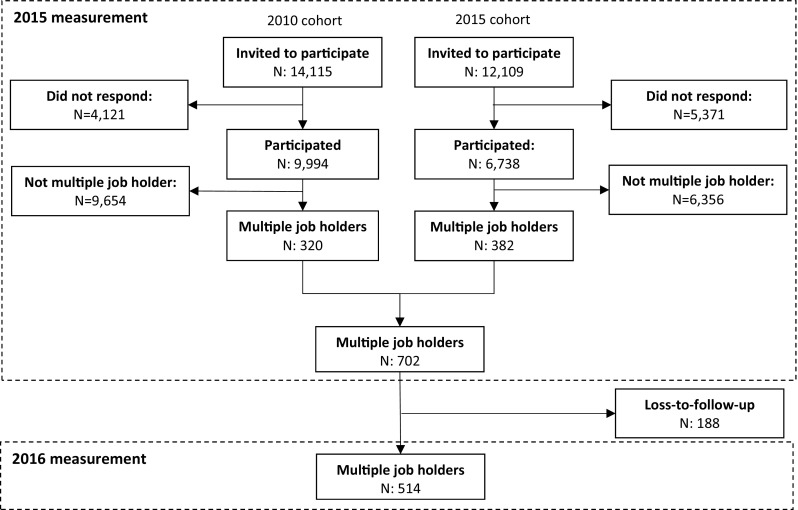
Study population

In total, 702 multiple job holders were included in this study, of which 188 were lost during follow-up (see Fig. 1). In Table 3, the characteristics of these multiple job holders are shown.

The results of the LCA, used to identify distinct groups of multiple job holders, are shown in Table 2. Although model 5 had a significant BLRT, indicating that it was an improvement over model 4, we chose model 4, because it was the last model to show an improvement in the BIC. In addition, the posterior probabilities of the groups in model 4 were, on average, higher than in model 3 and model 5. All solutions yielded useful and interpretable results.

**Table 2:** Results of latent class analysis

	Bayesian Information Criterion	Posterior probabilities	Number of respondents in each category	Bootstrap LRT
Model 1	38221.113	1. 1.000	1. 702	–
Model 2	37505.500	1. 0.926 2. 0.939	1. 296 2. 406	0.000
Model 3	37296.641	1. 0.872 2. 0.916 3. 0.921	1. 195 2. 170 3. 337	0.000
*Model 4*	*37266.418*	*1. 0.939* *2. 0.955* *3. 0.913* *4. 0.877*	*1. 113* *2. 310* *3. 145* *4. 134*	*0.000*
Model 5	37334.027	1. 0.874 2. 0.916 3. 0.840 4. 0.933 5. 0.873	1. 138 2. 108 3. 113 4. 243 5. 100	0.000

The model in italics is the model we selected

Table 3 presents the characteristics of the four distinct groups of multiple job holders: vulnerable multiple job holders, indifferent multiple job holders, satisfied hybrid multiple job holders, and satisfied combination multiple job holders. In the vulnerable multiple job holder group, the most common reason for MJH was financial necessity (37%). These workers were less satisfied with their job(s) than other multiple job holders (60% was satisfied), and experienced few benefits and many disadvantages of having multiple jobs. Many of them preferred having one job (81%). The multiple job holders in this group had relatively demanding jobs, while their jobs provided them with little autonomy (40% reported low autonomy in both jobs). In addition, they felt relatively less able to change their life and work (average mastery in this group was 3.3 compared to 3.7, 4.0, and 4.1 in the other groups). In addition, a relatively large proportion of these multiple job holders experienced financial difficulties (36% compared to 25%, 12%, and 7% in the other groups).

**Table 3:** Description of study population and four groups of multiple job holders

	All multiple job holders	Vulnerable multiple job holders	Indifferent multiple job holders	Satisfied hybrid	Satisfied combination
Number of respondents	702	145	134	310	113
*Reason for MJH*					
Reason for MJH					
Impossible to work more hours at current job	10%	19%	27%	1%	4%
Work more hours to make ends meet	15%	37%	22%	5%	5%
To earn some extra money	12%	13%	9%	14%	8%
To retain income security	7%	11%	5%	5%	7%
To start a business	9%	1%	0%	19%	0%
To get experience in another job	2%	2%	0%	2%	3%
Because of the variation	14%	5%	15%	16%	21%
Because I enjoy the combination of jobs	22%	5%	12%	28%	40%
Other	11%	7%	10%	11%	13%
Pro-active development (1–5)	3.9 (3.5–4.3)^a^	3.8^b^	3.5^b^	4.1^b^	4.1^b^
*Experience with MJH*					
Job satisfaction					
Unsatisfied	6%	16%	3%	5%	0%
Not satisfied, not unsatisfied	13%	24%	11%	11%	5%
Satisfied	81%	60%	86%	84%	95%
MJH history (in years)	7.4 (1.0–10.3) ^a^	6.4^b^	5.4^b^	8.4^b^	8.6^b^
Combining work schedules provides freedom					
No	46%	80%	34%	33%	48%
Yes	54%	20%	66%	67%	52%
Combining work schedules causes stress					
No	52%	9%	73%	61%	62%
Yes	48%	91%	27%	39%	38%
Combining demands of employers is difficult					
No	70%	27%	93%	77%	83%
Yes	30%	73%	7%	23%	17%
Doing work of job one in time of job two					
No	63%	60%	93%	54%	58%
Yes	37%	40%	7%	46%	42%
Learning skills in one job you do not learn in the other					
No	44%	56%	77%	29%	31%
Yes	56%	44%	23%	71%	69%
Performance one job worse because of other job					
No	83%	62%	100%	85%	88%
Yes	17%	38%	0%	15%	12%
Performance one job better because of other job					
No	43%	40%	84%	31%	33%
Yes	57%	60%	16%	69%	67%
Rather have one job? (yes)	39%	81%	38%	29%	10%
Job characteristics					
MJH type					
Combination	46%	65%	80%	0%	100%
Hybrid	54%	35%	20%	100%	0%
Contract type					
No permanent in both jobs	31%	26%	28%	23%	2%
Permanent in one job	55%	44%	32%	77%	39%
Permanent in both jobs	24%	30%	40%	0%	59%
Total working hours per week	39 (29–48)^a^	34^b^	27^b^	45^b^	43^b^
Working outside office hours (yes)	87%	87%	64%	94%	93%
Physical demands					
Low in both jobs	52%	23%	45%	70%	51%
High in one job	31%	44%	31%	23%	33%
High in both jobs	18%	33%	24%	8%	16%
Quantitative demands					
Low in both jobs	38%	27%	63%	38%	26%
High in one job	41%	41%	27%	46%	43%
High in both jobs	21%	32%	10%	17%	31%
Autonomy					
Low in both jobs	19%	39%	21%	5%	30%
High in one job	40%	46%	36%	40%	35%
High in both jobs	41%	15%	43%	56%	35%
Ability to change life and work					
Able to find new employer in next 12 months?					
No	33%	35%	43%	30%	24%
Maybe	36%	41%	39%	32%	36%
Yes	31%	24%	18%	37%	39%
Mastery (1–5)	3.8 (3.4–4.1)^a^	3.3^b^	3.7^b^	4.0^b^	4.1^b^
Social factors					
Providing informal care (yes)	32%	33%	36%	30%	34%
Partner (yes)	75%	65%	76%	79%	75%
Work-family conflict (yes)	60%	66%	28%	68%	68%
Financial factors					
Household financial situation					
Short of money	20%	36%	25%	12%	7%
Just adequate	24%	37%	32%	20%	22%
Money left	57%	28%	58%	68%	71%
Breadwinner (yes)	61%	57%	44%	68%	69%
Demographics (not included in latent class analysis)					
Male	45%	31%	28%	58%	51%
Educational level					
Low	20%	26%	38%	12%	10%
Medium	34%	41%	42%	25%	37%
High	47%	33%	20%	63%	53%
Age (years)					
45–49	39%	37%	36%	39%	45%
50–54	24%	27%	22%	25%	21%
55–59	17%	23%	17%	13%	20%
60–64	14%	10%	15%	17%	12%
65–70	6%	2%	10%	6%	3%
Health (not included in latent class analysis)					
Physical health (0–100)					
Baseline	51.7	49.6	52.4	52.0	52.9
One-year follow-up	51.4	49.4	52.4	51.6	52.1
Mental health (0–100)					
Baseline	52.4	48.8	53.9	52.7	54.4
One-year follow-up	51.7	48.6	53.5	51.6	54.0

^a^Average and inter quartile range

^b^Average

The most common reasons for MJH in the indifferent multiple job holder group was hours constraint in the first job (27%), and making ends meet (22%). A large majority (86%) was satisfied with their job, and experienced few benefits or disadvantages of having multiple jobs. About 60% of the multiple job holders in this group preferred having multiple jobs. On average, they worked few hours (27 per week), and relatively often in jobs with relatively low demands (45% reported low physical demands in both jobs, and 63% low quantitative demands in both jobs) and average autonomy. Their ability to change life and work was average, as well as the household financial situation (25% was short of money, and 54% had money left).

The satisfied hybrid multiple job holder group consisted solely of respondents who were self-employed in their second job. This group often had multiple jobs, because they enjoyed the combination of jobs (28%), wanted to start a business (19%) or because of the variation resulting from MJH (16%). They were satisfied with their job(s) (84%), and experienced benefits of combining jobs. A majority preferred having multiple jobs to having one job (71%). They worked many hours (45 per week), and many worked outside office hours (94%), but experienced low levels of job demands (70% and 38% reported low physical and quantitative demands in both jobs, respectively) and high levels of autonomy (56% reported high autonomy in both jobs). They also generally felt able to change their life and work, and often had money left (68%).

Multiple job holders in the satisfied combination multiple job holders all had a second job as an employee. They mainly had multiple jobs, because they enjoyed the combination of their jobs (40%). The vast majority was satisfied with their job(s) (95%) and experienced benefits of combining jobs. Regarding the other characteristics, multiple job holders in this group were similar to multiple job holders in the satisfied hybrid group. A large majority preferred having multiple jobs over having a single job (90%). About 60% had a permanent contract in both jobs, and on average, they worked 43 h per week, and many outside office hours. Relatively many multiple job holders in this group reported high quantitative demands in both jobs (31%), while the percentage reporting high physical demands and autonomy in both jobs was average (16% and 35%, respectively). Furthermore, they felt relatively able to change their lives and work. Many multiple job holders in this group were breadwinner (69%) and had money left (71%).

Regarding demographic factors, men were overrepresented in the satisfied hybrid and the satisfied combination groups, whereas women were overrepresented in the vulnerable and indifferent groups. Persons with a low educational level were especially overrepresented in the indifferent group, while they were underrepresented in the satisfied hybrid and satisfied combination groups. No large differences were found between the four groups regarding age.

At baseline, we found that vulnerable multiple job holders experienced significantly poorer physical and mental health than the other groups of multiple job holders (see Tables 4, 5). No significant difference in change in physical and mental health after 1 year of follow-up was found. No significant effect modification of gender on the relation between MJH group and health was found. Age significantly modified the relation between satisfied hybrid and mental health at baseline and during follow-up. Stratified analyses showed that at baseline, health differences between vulnerable multiple job holders and hybrid multiple job holders were larger among workers aged 55 years and older than among workers aged 45–54 years. During follow-up, no significant differences in changes health were found between groups of multiple job holders in stratified analyses (data not shown).

**Table 4:** Association between MJH and physical health

	Baseline				1-year follow-up			
	Crude		Adjusted^a^		Crude^b^		Adjusted^c^	
	*B*	95% CI	*B*	95% CI	*B*	95% CI	*B*	95% CI
MJH group								
Vulnerable	–	–	–	–	–	–	–	–
Indifferent	2.86*	1.02–4.71	3.09*	1.23–4.94	1.43	− 0.35 to 3.21	0.21	− 1.51 to 1.93
Satisfied combination	3.33*	1.42–5.23	2.91*	0.96–4.85	1.04	− 0.81 to 2.88	0.87	− 1.02 to 2.76
Satisfied hybrid	2.43*	0.89–3.96	1.86*	0.24–3.47	1.31	− 0.15 to 2.77	0.42	− 0.93 to 1.78

*Significant at *α* < 0.05

^a^Adjusted for: gender, age, and educational level

^b^Adjusted for: physical health at baseline

^c^Adjusted for: physical health at baseline, gender, age, and educational level

**Table 5:** Association between MJH and mental health

	Baseline				1-year follow-up			
	Crude		Adjusted^a^		Crude^b^		Adjusted^c^	
	*B*	95% CI	*B*	95% CI	*B*	95% CI	*B*	95% CI
MJH group								
Vulnerable	–	–	–	–	–	–	–	–
Indifferent	5.11*	3.24–6.98	4.63*	2.79–6.48	1.70	− 0.54 to 3.93	1.71	− 0.51 to 3.93
Satisfied combination	5.61*	3.68–7.55	6.12*	4.19–8.05	1.54	− 0.78 to 3.86	1.33	− 1.02 to 3.69
Satisfied hybrid	3.89*	2.33–5.45	4.54*	2.93–6.15	0.27	− 1.56 to 2.10	− 0.21	− 2.15 to 1.74

*Significant at *α* < 0.05

^a^Adjusted for: gender, age, and educational level

^b^Adjusted for: mental health at baseline

^c^Adjusted for: mental health at baseline, gender, age, and educational level

## Discussion

The aims of this study were to empirically distinguish groups of multiple job holders and to analyze whether or not differences in health between these groups exist. We were able to identify four groups of multiple job holders: (1) vulnerable multiple job holders; (2) indifferent multiple job holders; (3) satisfied hybrid multiple job holders; and (4) satisfied combination multiple job holders. At baseline, vulnerable multiple job holders experienced worse physical and mental health than the other groups. We found no significant differences between the groups regarding changes in physical or mental health after 1 year of follow-up.

Our findings are partly in line with the profiles conceptualized, but not empirically tested, by Rouault (2002). The vulnerable group we found closely resembles the proletarian survivor profile identified by Rouault. This group consists of workers who have multiple low-quality jobs out of financial necessity. The satisfied combination and satisfied hybrid group we identified are similar to the ideal-type profile distinguished by Rouault. These multiple job holders enjoy their jobs and experience benefits and few disadvantages from MJH. Their households are financially well-off and they feel in charge of their life and work. The indifferent group we identified, consisting of workers who experience few benefits or disadvantages of MJH, was not conceptualized by Rouault. A possible explanation is that in much previous research, it is assumed that the decision to take on multiple jobs is based on careful consideration of the (financial) advantages and disadvantages (Panos et al. 2014; Conway and Kimmel 1998). However, our previous qualitative study showed that the decision to take on multiple jobs is not well-considered by some multiple job holders (Bouwhuis et al. 2018). Another explanation may be that this group, characterized by relatively low number of working hours (27 per week compared to 39 in the total study population) is specific to The Netherlands, where part-time work is more common than in other countries, especially among women (Roeters and Craig 2014).

We identified one group that we labeled vulnerable multiple job holders, because this group combined precarious work and employment conditions with adverse personal characteristics, such as poor household financial situation (Burgess et al. 2013). Many of these multiple job holders reported stress as a result of conflicting work schedules. In addition, many of them had high job demands and low autonomy in both jobs. This may result in a deterioration of health and reduced sustainable employability (Bakker and Demerouti 2007). More flexible work schedules and a better balance between job demands and resources may prevent adverse health consequences and improve sustainable employability among this group of older multiple job holders. Awareness among employers of other jobs and work schedules as well as associated demands and resources may contribute to this. In addition, vulnerable multiple job holders  had lower levels of mastery and worse household financial situation than multiple job holders in other groups. Previous qualitative research has suggested that feelings of mastery and a favorable financial situation are important resources for multiple job holders, enabling them to change their work situation, e.g., find one or more different jobs, if they are dissatisfied (Bouwhuis et al. 2018). Policies aimed at increasing these resources, e.g., life-long learning (Van Der Heijden et al. 2009), may improve sustainable employability among vulnerable multiple job holders, since it may provide them with skills and self-efficacy needed to find a different job.

We found health differences between groups of multiple job holders at baseline: vulnerable multiple job holders experienced worse physical and mental health than the other groups. This finding may contribute to explaining the mixed findings of the previous research regarding the association between MJH and health (Jamal et al. 1998; Bouwhuis et al 2017a; Marucci-Wellman et al. 2014, 2016). The distribution of multiple job holders over different groups can differ per country or over time, which may influence the overall association between MJH and health. Therefore, we recommend that future research on the association between MJH and health takes the heterogeneity of multiple job holders into account.

We did not find a statistically significant difference in changes in health. One explanation could be that health status is a predictor of MJH, rather than an outcome (Jamal et al. 1998). Previous research using the same cohort, however, found that health status did not contribute to predicting transitions from single job holding to multiple job holding (Bouwhuis et al. 2017b). However, that study did not distinguish between subgroups of multiple job holders. It is possible, for instance, that poor health predicts vulnerable MJH, whereas good health predicts satisfied combination MJH. Another explanation may be that the present study was conducted among older workers, who most likely have already been exposed to MJH and job demands and resources for a long time. One year of follow-up may not be long enough for significant changes in health to occur in this group.

No significant differences in health were found between men and women in the different MJH groups, though health differences have been reported in the general population (Denton et al. 2004). Men and women were not equally distributed across the MJH groups, e.g., 69% of the workers in the vulnerable group were female and 72% of the vulnerable group, compared with 55% in the whole study population. Possibly, the variables used to distinguish MJH groups also (partly) explain gender differences in health.

A main strength of this study is the quality of the data we used to identify groups; it contained very few missing values. In addition, we included many variables in the LCA to distinguish groups, enabling us to construct comprehensive profiles. Another strength is that we were able to study health at baseline as well as changes in health after 1 year of follow-up. This study also has limitations. First, we lost 188 respondents to follow-up. However, we found no significant differences between the respondents who participated in 2015 and 2016 (*N* = 514) and those lost to follow-up regarding gender, age, educational level, the group in which they were classified, or health. Furthermore, as we included participants in the fifth wave of STREAM, selective loss to follow-up may have occurred before baseline, i.e., multiple job holders with poorer health may have been more likely to drop out of STREAM before the fifth wave or they may have quit MJH. However, we did not find differences regarding health, MJH type, and job satisfaction between those who were lost to follow-up before the fifth wave, or those who no longer had multiple jobs in the fifth wave, and those who still had multiple jobs in the fifth wave. Besides, respondents of STREAM are part of an internet panel, which may have caused an underrepresentation of groups with limited access to internet. This may have biased our findings (Bethlehem 2010). In addition, we used USA weights to calculate sum scores of the physical and mental component of the SF-12. Using standard weights may have influenced our findings, since a previous study has found that using standard scoring methods may result in altered correlations between the SF-12 physical and mental component summary scores as well as external variables such as age (Hagell et al. 2017). Furthermore, using USA weights to calculate sum scores of the physical and mental component of the SF-12 in our Dutch population may have influenced our findings. However, previous research has shown that weights based on a Dutch population were similar to the USA weights (Mols et al. 2009). Therefore, we think that this did not strongly affect our findings. Furthermore, this study was conducted among Dutch workers aged 45 years and older in The Netherlands and did not include workers who were self-employed in all jobs. Inclusion of younger multiple job holders or those who were self-employed in all of their jobs may have resulted in other groups. Furthermore, it is possible that a similar study in a different country would result in different groups of multiple job holders. For instance, differences in social security systems may influence the reasons for and experiences with MJH. Extensive social security systems may result in relatively lower numbers of workers who have multiple jobs for financial reasons, for example.

In conclusion, four different groups of older multiple job holders could be distinguished: (1) vulnerable multiple job holders; (2) indifferent multiple job holders; (3) satisfied hybrid multiple job holders; and (4) satisfied combination multiple job holders. At baseline, vulnerable multiple job holders experienced worse physical and mental health. We found no statistically significant differences between groups of multiple job holders regarding changes in health after 1 year of follow-up. We recommend that future research on MJH takes into account these distinct groups. To support vulnerable multiple job holders, employers are recommended to increase flexibility to prevent conflicts between work schedules and policy makers are recommended to stimulate life-long learning to enable vulnerable multiple job holders to find higher quality jobs that match their preferences.
